# Navigating the Dry Eye Therapeutic Puzzle: A Mechanism-Based Overview of Current Treatments

**DOI:** 10.3390/ph18070994

**Published:** 2025-07-02

**Authors:** Jason Betz, Anat Galor

**Affiliations:** 1 Bascom Palmer Eye Institute, University of Miami, Miami, FL 33136, USA; jasondbetz0@gmail.com; 2 Surgical Services, Miami Veterans Administration Medical Center, Miami, FL 33125, USA

**Keywords:** dry eye disease, aqueous tear deficiency, meibomian gland dysfunction, ocular pain, neuropathic pain

## Abstract

**Background/Objectives**: Dry eye disease (DED) is a multifactorial condition with complex pathophysiology involving tear film instability, ocular surface inflammation, and nerve dysfunction. This review summarizes current evidence on the different available therapies targeting these mechanisms. **Methods**: A review of clinical studies evaluating treatment outcomes for therapies targeting aqueous tear deficiency, Meibomian gland dysfunction, ocular surface inflammation, and ocular pain was conducted, with an emphasis on randomized controlled trials and meta-analyses where available. **Results**: Artificial tears provide symptomatic relief with limited impact on tear film stability. Punctal plugs improve tear retention but show variable efficacy across studies. Treatments targeting MGD—such as lipid-based lubricants, eyelid hygiene, thermal pulsation (LipiFlow, iLux), and intense pulsed light (IPL)—demonstrate improvements in gland function, though outcomes vary. Anti-inflammatory agents including cyclosporine, lifitegrast, and short-term corticosteroids improve ocular surface signs, with mixed symptom relief. Biologic therapies like autologous serum tears and platelet-rich plasma show promise for both signs and symptoms, but data remain inconsistent. Nerve-targeted therapies, including oral neuromodulators (gabapentin, antidepressants), botulinum toxin, and transcutaneous nerve stimulation, have shown potential for managing neuropathic ocular pain, although randomized data are limited. Overall, variability in study designs, patient populations, and outcome measures highlights the need for more rigorous research. **Conclusions**: Personalized, mechanism-based treatment strategies are essential for optimizing outcomes in DED. Future research should prioritize well-designed, controlled studies to clarify the role of emerging therapies and guide the individualized management of this heterogeneous condition.

## 1. Introduction

### 1.1. What Is Dry Eye Disease (DED)?

Dry eye disease (DED) is “a multifactorial disease of the ocular surface characterized by a loss of homeostasis of the tear film, and accompanied by ocular symptoms, in which tear film instability and hyperosmolarity, ocular surface inflammation and damage, and neurosensory abnormalities play etiological roles” [1]. Various pathologies can contribute to the DED presentation including ocular pathologies (e.g., lacrimal gland, Meibomian gland, or goblet cell dysfunction), systemic diseases (e.g., Sjögren’s disease (SjD), graft-versus-host disease (GVHD), fibromyalgia, migraine), medications (e.g., anti-histamines, antidepressants), and environmental factors (e.g., air pollution, smoking, sleep) [2,3,4], making the selection of the optimal therapy challenging. This is especially important as DED is common, affecting 5% to 50% of the global population [2]. As the understanding of DED evolves, so does the therapeutic landscape, necessitating a nuanced approach to treatment tailored to individual patient profiles.

### 1.2. How Does DED Present?

In terms of presentation, DED can present with a variety of pain- and non-pain-related symptoms including descriptors such as dryness, burning, grittiness, tearing, foreign body sensation, and blurred vision. Signs of DED are likewise varied and include nociceptive contributors such as aqueous tear deficiency (ATD), tear film instability, epithelial disruption, and/or inflammation, which can be variably present in individuals [5]. Additional nociceptive factors that can influence the DED presentation include an abnormal anatomy of the eyelid, conjunctiva, and cornea (e.g., lid laxity, ectropion, lagophthalmos, fibrosis, conjunctivochalasis, pterygium, Salzmann); organ dysfunction (e.g., in Meibomian glands or conjunctival goblet cells); and anterior blepharitis [3,6,7].

### 1.3. How Is DED Profiled?

DED symptoms can be captured using a variety of composite questionnaires. For example, the 5-Item Dry Eye Questionnaire 5 (DEQ5) quantifies dryness, discomfort, and tearing frequency/intensity [8], while the Ocular Surface Disease Index (OSDI) quantifies symptoms (soreness, poor vision), triggers (wind, light), and the impact of symptoms on the quality of life (QoL) [9].

Signs of tear dysfunction can be assessed during a slit lamp exam and with adjunct point-of-care tests and imaging modalities. Specifically, tear film stability is measured by tear break-up time (TBUT), conjunctival and corneal epithelial disruption with the help of Lissamine green or fluorescein staining, tear production with Schirmer strips, Meibomian gland function by inspecting the eyelid margin and expressing meibum, and anatomical factors by evaluating eyelid laxity and conjunctivochalasis [10,11]. Point-of-care tests such as InflammaDry (Quidel, San Diego, CA, USA) assess for the presence of ocular surface inflammation, and tear osmolarity testing (TearLab, Escondido, CA, USA) examines tear osmolarity [10]. Lastly, imaging modalities (e.g., Meibography and Keratograph (Oculus, Wetzlar, Germany)) provide information on gland structure, tear film break-up time, tear meniscus height, and lipid layer thickness. Together, these tools provide a comprehensive assessment of ocular surface status, aiding in the classification of DED subtypes and guiding targeted management strategies.

### 1.4. The Role of Nerve Dysfunction in DED

Beyond nociceptive sources, neurotrophic, neuropathic, and nociplastic etiologies may also contribute to the DED presentation [12,13,14]. In addition to normally functioning nociceptors producing a physiologic pain response to noxious stimuli and cellular injury (e.g., tear hyperosmolarity, environmental irritants), peripheral, central, and autonomic nerves may become dysfunctional and influence the DED presentation.

First, corneal nerves may have a loss of sensation. When accompanied by corneal pathology (staining, epithelial defect, corneal melt), the entity is called neurotrophic keratitis (NK) [15]. Due to inappropriate sensing, NK often leads to low tear production and epithelial disruption, underscoring its presence as a contributor to DED signs. In fact, NK often presents with DED signs that are out of proportion to symptoms.

**Figure 1:**
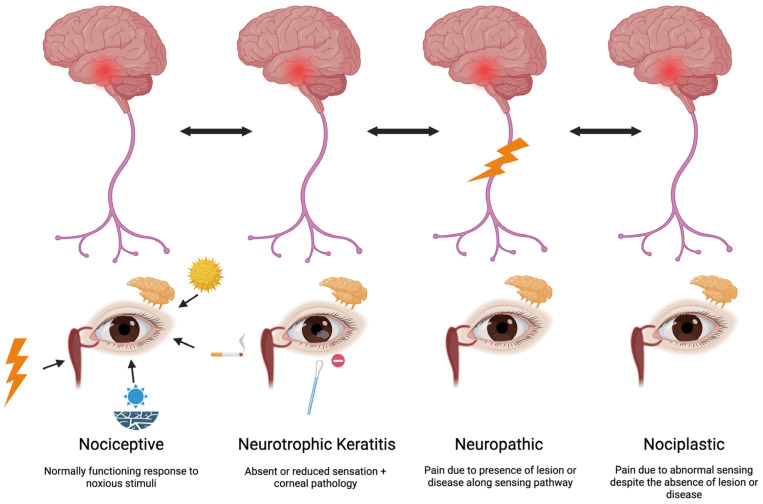
Nerve dysfunction in DED. This schematic outlines the role of peripheral and central nerve pathways in the pathophysiology of DED. Nociceptive pain arises from the appropriate activation of corneal nociceptors in response to tear film instability, ocular surface damage, or environmental irritants. Neurotrophic keratitis is a distinct degenerative pathology of the corneal epithelium resulting from impaired corneal nerves. Neuropathic mechanisms result from nerve injury or dysfunction, leading to aberrant signaling. Nociplastic pain arises from altered neural processing despite no clear evidence of tissue damage or disease. These distinct but overlapping pathways contribute to symptom and sign variability in DED and highlight the need for targeted, mechanism-specific therapies.

In contrast, nerve dysfunction can present in other ways, namely, when a neuropathic or nociplastic component underlies pain, the DED presentation is one of the symptoms that outweigh signs [16]. The entity is termed neuropathic when it is due to the presence of a lesion or disease and nociplastic when no lesion or disease is identified [17]. Both entities lead to the inappropriate transmission of pain signals, even in the absence of typical pain-inducing stimuli. This dysfunction may underlie the frequently observed discordance between patient-reported symptoms and clinical signs of tear dysfunction [18,19]. Often, not one but a combination of mechanisms (e.g., nociceptive, neurotrophic, neuropathic, nociplastic) contributes to symptoms and/or signs of DED (Figure 1) [20].

### 1.5. How Is Nerve Status Profiled?

Corneal sensation testing can be performed qualitatively using a cotton swab, dental floss, or tissue paper or quantitatively using devices like the Brill, Cochet-Bonnet, or Belmonte esthesiometers [21,22,23,24,25]. Qualitative methods allow for the rapid assessment of absent, low, normal, or hyper-corneal sensation but are subject to provider interpretation. Alternatively, quantitative assessment allows for a more precise analysis of sensation but is typically limited to research settings and the availability of devices. The anesthetic challenge is another test performed to understand nerve status. The challenge involves assessing pain pre and post application of a topical anesthetic (e.g., 0.5% proparacaine hydrochloride) to the ocular surface to differentiate between peripheral versus central or non-ocular sources of corneal pain. The complete relief of pain following anesthetic suggests a peripheral origin to pain (nociceptive or peripheral neuropathic), while persistent pain indicates a central or non-ocular surface source of pain or a mixed etiology [26]. The presence of cutaneous allodynia (increased pain/sensitivity to light touch around the periorbital region) also suggests a central mechanism to pain [27]. In vivo confocal microscopy (IVCM) may be a useful adjunct tool for assessing corneal structures at the cellular level [28]. Nerve anatomy characteristics such as density, tortuosity, and anatomy can be captured, with some studies suggesting that morphologically defined microneuromas can be a potential biomarker of peripheral neuropathic pain [29]. The presence of corneal immune cells (e.g., activated dendritic cells) in the central cornea indicate that inflammation at the level of the corneal nerves may contribute to the DED presentation. Together, these tools help provide a comprehensive assessment of nerve function, aiding in the classification of DED subtypes and guiding targeted management strategies.

### 1.6. Review Objective

The objective of this review is twofold: to evaluate the efficacy and mechanisms of current therapeutic options and to offer therapy recommendations based on the type of DED, as well as patient-specific factors such as comorbid conditions. Building on the existing literature, this review synthesizes current evidence through a comprehensive, mechanism-based lens by integrating therapies targeting aqueous deficiency, Meibomian gland dysfunction, and inflammatory and nerve pathways. By organizing treatments according to DED subtype and underlying pathophysiology, and by addressing emerging modalities such as novel topical formulations, blood products, and neuromodulation, this review provides clinicians with a practical, precision-guided framework that extends beyond conventional management strategies (Table 1).

## 2. Review of Current Therapies

**Figure 2:**
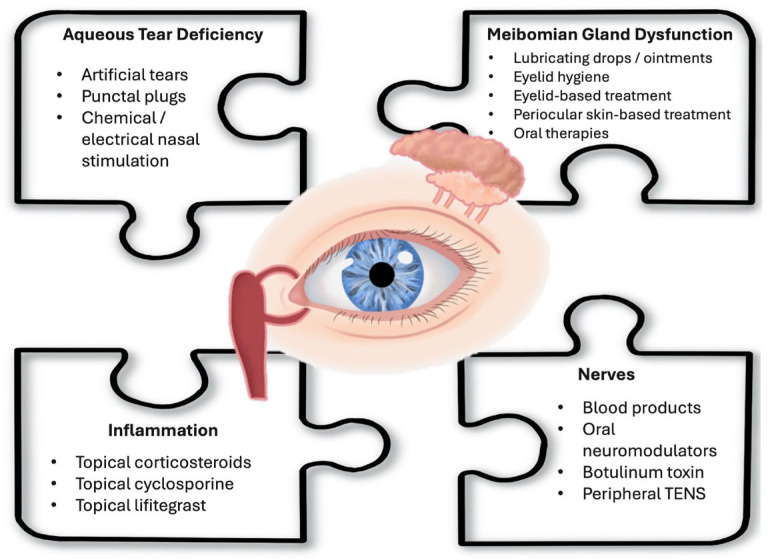
An overview of dry eye disease and treatment targets. This diagram illustrates the key mechanisms contributing to dry eye disease (DED), including aqueous tear deficiency (ATD), Meibomian gland dysfunction (MGD), ocular surface inflammation, and nerve dysfunction. The treatment options discussed in this review are mapped to their respective targets within these pathways, highlighting the importance of a mechanism-based, individualized approach to DED management. TENS = transcutaneous electrical nerve stimulation.

In the following sections, this review will methodically examine therapies for DED, categorized according to the primary subtype they target: ATD, tear film instability, inflammation, and nerve-mediated mechanisms (Figure 2). While these classifications facilitate a structured discussion of each therapeutic approach, it is crucial to acknowledge that the effective management of DED in clinical practice requires a personalized strategy. Individual patient treatment plans often necessitate a stepwise approach, integrating multiple therapies to address the complex interplay of ATD, tear film instability, inflammatory processes, and nerve contributions. This approach aligns with current perspectives that recommend sub-classifying DED based on predominant mechanisms while recognizing that aqueous deficient and evaporative forms frequently coexist along a pathophysiologic continuum while also acknowledging cases where signs and symptoms are discordant [1].

### 2.1. Therapies for Aqueous Tear Deficiency

Aqueous tear deficiency in DED results from the inadequate production of the aqueous component of tears by the lacrimal glands and accessory lacrimal glands. This can be a result of lacrimal gland hypofunction due to gland destruction or inappropriate sensing/innervation [30,31], as can be seen in systemic diseases (SjD, GVHD), or less commonly secondarily due to conjunctival cicatrization or the acquired or congenital loss of the lacrimal gland [32]. Schirmer test results are often used to diagnose ATD, with lower values indicating more severe disease. Addressing ATD involves strategies to supplement or conserve the tear film. The following highlights some common therapies.

#### 2.1.1. Artificial Tears

Artificial tears (ATs) are cornerstones of managing ATD by providing symptomatic relief and supplementing the aqueous layer of the tear film. These over-the-counter lubricants mimic the properties of natural tears and are available in various formulations, including solutions with differing viscosities and osmolarities to cater to the specific needs of patients. Multiple systematic reviews have demonstrated improvement in DED signs and symptoms after regular AT use [33,34]. In one randomized control trial of 188 patients receiving standard formula 1.0% carboxymethlycelluose versus carboxymethylcellulose 1.0% and glycerin 0.9%, OSDI scores progressively improved from baseline to day 30 in both groups (*p* < 0.001 for both groups) [35]. These findings highlight the efficacy of artificial tears, in multiple formulations, in alleviating dry eye symptoms, reinforcing their role as a first-line therapy in the management of DED.

#### 2.1.2. Punctal Plugs

Punctal plugs are used to retain tears on the ocular surface by blocking the tear drainage through the puncta. This method increases the eye’s tear retention and maintains surface moisture [36]. Punctal plugs are available in various forms, categorized by material composition and resting site within the lacrimal drainage system. Temporary plugs, composed of absorbable materials such as collagen or other synthetic materials, degrade over time, while permanent plugs, typically made of silicone or acrylic, provide long-term occlusion. These devices can be left at the level of the punctum or advanced into the canaliculus of the lacrimal drainage system [37]. In one study, 37 individuals with DED (Schirmer test < 5 mm or TBUT < 5 s, presence of any DED symptoms, staining with fluorescein or Rose Bengal) had atelocollagen absorbable plugs placed. Schirmer test values increased from baseline to 8 weeks after placement (3.9 ± 0.5 to 6.0 ± 0.5 mm, *p* < 0.05), while OSDI scores decreased (55.5 ± 0.9 to ~30.0 ± 0.1 at 8 weeks, *p* < 0.05). In fact, OSDI scores remained significantly decreased compared to baseline at all time points, 1 week, 4 weeks, 8 weeks, 16 weeks, and 24 weeks after plug placement (*p* < 0.05) [38]. While these findings suggest clinical benefit in select populations, broader evidence remains mixed. In a systematic review of 18 studies involving 711 participants, considerable variability in outcomes was reported, with inconsistent data on the degree of symptom and tear film improvement across studies. The review also found no clear advantage among different plug materials, including silicone, collagen, and acrylic. Additionally, methodological limitations such as small sample sizes, varying follow-up periods, and inconsistent outcome measures further limit the generalizability of these findings [37]. Together, these findings illustrate variability in the reported efficacy of punctal plugs across studies; however, their ability to enhance tear retention suggests they may remain a useful option for managing ATD. Further well-designed randomized controlled trials are needed to clarify their therapeutic role and optimize clinical outcomes.

#### 2.1.3. Tyrvaya (Varenicline Solution)

Neurostimulation for ATD targets the lacrimal functional unit, comprising the corneal and conjunctival epithelium, lacrimal glands, Meibomian glands, and conjunctival goblet cells [39]. Tyrvaya nasal spray (varenicline solution 0.03 mg; Oyster Point Pharma Inc., Princeton, NJ, USA), approved in 2021, is a selective nicotinic acetylcholine receptor agonist targeting the trigeminal parasympathetic pathway to achieve this goal [40]. In a phase 3 randomized control trial, 758 individuals with mild or greater ATD (Schirmer ≤ 10, AT use, OSDI ≥ 23) received either twice-daily treatment with 50 μL intranasal spray in each nostril of varenicline solution (0.06, *n* = 246; 0.03 mg *n* = 260) or vehicle (*n* = 252) for 4 weeks. Schirmer scores more frequently improved by ≥10 mm in the 0.06 mg and 0.03 mg varenicline groups compared to control (49% vs. 47% vs. 28%, *p* < 0.0001). Eye dryness scores also improved from baseline at four weeks in the 0.06 mg and 0.03 mg varenicline groups (least square (LS) mean difference from vehicle: −6.8, *p* = 0.001 and −4.4, *p* = 0.04, respectively) [41]. This therapy offers a promising alternative for patients who may not respond adequately to traditional treatments such as artificial tears or punctal plugs.

#### 2.1.4. Nasal Stimulation—External Nasal Stimulator

Neurostimulation can also be achieved with electrical stimulation [39]. Nasal stimulation devices, such as iTear (Olympic Ophthalmics, Issaquah, WA, USA), approved in 2020, uses sonic external neurostimulation applied to the side of the nose to target the external nasal nerve (a branch of the trigeminal nerve). This approach has been shown to increase tear production and may influence goblet cell and Meibomian gland function [42,43]. In a clinical trial of 101 individuals with mild or greater ATD (anesthetized Schirmer ≤ 10), twice or more daily use of iTear external nasal stimulation resulted in increased tear production (change from unstimulated to stimulated tear production 9.4 mm, 95% CI 7.4–11.3, *p* < 0.05) and decreased OSDI scores (mean decrease −14.4, 95% CI −17.7–−11.1, *p* < 0.05) 30 days after baseline [42]. TrueTear (Allergan, Irvine, CA, USA), a neurostimulatory device that delivered a current to the inner nasal mucosa, demonstrated comparable efficacy in stimulating tear production [44]. However, it is no longer commercially available. These findings highlight the effectiveness of electrical devices, applied both external and internally, in increasing tear production and improving the symptoms of ATD, supporting their role as a non-invasive therapeutic option for DED.

### 2.2. Therapies for Meibomian Gland Dysfunction (MGD)

Like DED, MGD is an umbrella term comprising multiple phenotypes (e.g., eyelid keratinization, change in meibum quality, gland atrophy), each of which may variably contribute to tear film instability and DED symptoms. MGD can be assessed through a combination of clinical exam findings and adjunctive diagnostic tools. On exam, evaluation includes the inspection of the eyelid margin for evidence of telangiectasia, gland capping, and keratin. Meibomian gland expression and the quality of meibum can be graded to assess overall gland function. Additionally, imaging tests such as Meibography and Keratograph can be used to visualize gland structure and dropout [45]. Several therapeutic approaches have been used to target aspects of MGD and associated disorders (e.g., anterior blepharitis), including lubricating drops, lid hygiene, device-based treatments, and supplements.

#### 2.2.1. Lubricating Drops and Ointments

Lubricating eye drops and ointments may also improve tear film stability. As mentioned above, lubricating drops are available in a variety of differing viscosities and formulations. Some of these products target lipid abnormalities by adding liposomes, lipid emulsions, and other lipid-based carrier compounds, with the potential to stabilize the tear film [46]. One study of 210 individuals with MGD-associated DED (MGD grade ≤ 2 for meibum expressibility and quality, TBUT ≤ 5 s, and unanesthetized Schirmer ≥ 3) randomized individuals to a lipid-based lubricant eye drop formulation (hydroxypropyl guar/propylene glycol/phospholipid) versus preservative-free saline eye drops. The lipid-based drop significantly increased TBUT from baseline compared to the saline drop (mean difference 1.0 ± 0.3 s, 95% CI 0.4–1.6, *p* = 0.001). Additionally, symptoms improved to a greater degree in the lipid versus saline drop (Impact of Dry Eye on Everyday Life questionnaire score mean difference 16.0 ± 3.6, 95% CI 8.9–23.1, *p* < 0.0001) [47]. These findings underscore the efficacy of lipid-based lubricating eye drops in enhancing tear film stability and alleviating symptoms in patients with MGD-associated DED, highlighting their role as a targeted therapy option. Looking ahead, experimental delivery models, such as novel copolymers that chemically adhere to the ocular surface, are also being investigated for enhanced drop retention and efficacy [48].

#### 2.2.2. Perfluorohexyloctane Ophthalmic Solution (MIEBO)

Perfluorohexyloctane ophthalmic solution, branded as MIEBO (Bausch + Lomb Americas Inc., Bridgewater, NJ, USA), and approved in 2023, is thought to stabilize the tear film by integrating with the lipid layer [49]. In a randomized control trial, 312 patients with MGD-associated DED (total MGD score of ≥3, TBUT ≤ 5 s, total corneal fluorescein staining (tCFS) > 4 and <11, unanesthetized Schirmer > 5, and OSDI ≥ 25) were randomized to receive perfluorohexyloctane or hypotonic (0.6%) saline drops four times daily. Notably, tear film stability remained unchanged at day 57, as there were no significant differences in TBUT or MGD score between treatment groups. However, perfluorohexyloctane-treated patients demonstrated significantly greater improvement compared to the hypotonic saline group in both the tCFS score (mean change: −1.14, 95% CI −1.70–−0.57, *p*  <  0.001) and eye dryness score (mean change: −12.74, 95% CI −17.20–−8.28, *p*  <  0.001) [50]. These data demonstrate the efficacy of perfluorohexyloctane in improving ocular surface staining and reducing symptom severity in patients with MGD-associated DED, reinforcing its role as a pharmacologic option in tear film instability DED. However, these data suggest that more studies are needed to understand the mechanisms that underlie the noted improvements.

#### 2.2.3. Eyelid Hygiene

The cleaning of the eyelids with appropriate solutions or wipes helps manage MGD by removing debris, bacteria, and excess oils that can clog the Meibomian glands. Eyelid hygiene practices have been shown to improve gland function and reduce symptoms related to MGD [51,52]. One trial of 60 individuals with grade 2 MGD (TFOS International Workshop on MGD definition [53]) randomized individuals to eyelid cleansing with OcuSOFT Lid Scrub Original Foaming Eyelid Cleanser or Johnson’s baby shampoo twice a day for 4 weeks. Both treatment groups demonstrated significant improvement in symptoms from baseline to 4 weeks (OSDI-Cleanser: 42.1 ± 14.0 to 17.9 ± 9.8, *p* < 0.001; baby shampoo: 42.6 ± 16.1 to 17.9 ± 9.8, *p* < 0.001), with no differences between the two groups [54]. Interestingly, MGD-related signs, including meibum quality, expressibility, and ocular staining, showed only mild improvement that did not reach statistical significance in either group. This study highlights the role of eyelid hygiene in improving dry eye symptoms; however, more studies are needed to understand the pathophysiological mechanisms behind its effect.

#### 2.2.4. Eyelid-Based Treatments

##### LipiFlow Thermal Pulsation System

Office-based procedures designed to improve Meibomian gland function aim to enhance gland secretion, reduce obstruction, and stabilize the tear film in patients with MGD. These treatments utilize heat, pulsation, and/or mechanical debridement, with the goal of alleviating gland obstruction and restoring tear film homeostasis [55,56]. The LipiFlow system (TearScience, Inc., or Johnson & Johnson, Morrisville, NC, USA), approved in 2011, is such a system that applies repeated, controlled heat and compression to the eyelids, specifically targeting the Meibomian glands to facilitate the expression of gland contents. Multiple systemic reviews highlight the variability in study design and results after LipiFlow treatment [57,58]. However, some studies found benefits of LipiFlow over at-home treatments. In a randomized control trial of 50 patients with MGD (Meibomian gland secretion (MGS) ≤ 12 in either eye, SPEED score ≥ 6), individuals receiving one LipiFlow treatment were compared to individuals performing daily lid hygiene with a wet cotton swab followed by 15 min warm compresses for two weeks. MGD signs 3 months after treatment improved in both groups, with significant improvements in TBUT (LipiFlow: 2.31  ±  0.96 to 5.58  ±  2.19 s, *p* < 0.01; warm compress: 2.67  ±  1.44 to 3.96  ±  1.89 s, *p* < 0.01; between groups *p* = 0.01) and MGS (LipiFlow: 8.84  ±  2.20 to 12.84  ±  3.92, *p* < 0.01; warm compress: 9.54  ±  3.10 to 10.24  ±  3.34, *p* = 0.3; between groups *p* = 0.04) in the LipiFlow versus warm compress group. Symptoms rated using the SPEED questionnaire decreased at 3 months in both groups compared to baseline (LipiFlow: 9.52 ± 2.88 vs. warm compress: 9.24 ± 2.02 to 3.84 ± 1.49 vs. 6.56 ± 2.77, *p* < 0.01) but again with a greater improvement in the LipiFlow groups (*p* < 0.01) [59]. Regarding treatment durability, a systematic review reported that improvements in symptoms and Meibomian gland function may persist for up to one year following a single LipiFlow session, although durability varied across studies depending on baseline severity, assessment metrics, and retreatment protocols [60]. These findings suggest that LipiFlow may offer sustained benefits beyond what is typically achieved with short-term home therapies, though additional long-term comparative trials are needed to better characterize the durability and optimal retreatment interval.

##### iLux

Another office-based treatment focused on Meibomian gland health is the iLux device (Alcon, Fort Worth, TX, USA), approved in 2017. iLux is a handheld device which delivers targeted heat and gentle pressure to the eyelids, with the goal of facilitating Meibomian gland secretion [61]. In a study of 130 patients with MGD (MGS < 15, TBUT < 10 s, OSDI ≥ 13), individuals were randomized to receive either a single iLux treatment or five sessions of manual expression. A total of 12 months after baseline, MGD signs, including MGS (β coefficient = 1.96, *p* < 0.001) and TBUT (β = 1.93, *p* < 0.001) and symptoms, including OSDI scores (β = −0.33, *p* < 0.001) were improved in iLux compared to manual expression [62]. While comparison data are limited, one randomized open-label trial of 142 individuals with MGD (MGS ≤ 12, TBUT < 10 s, and OSDI ≥ 23) comparing iLux versus LipiFlow found improvements in symptoms in both devices with no significant difference between them 4 weeks after treatment [61]. Taken together, in-office therapies, such as LipiFlow and iLux, have been shown to improve some clinical signs of MGD and decrease the symptoms of DED, and in some studies, they offered benefits over at-home procedures, though efficacy has varied across studies. These findings highlight the need for individualized treatment approaches and further research to optimize which approach is the best in each patient, with a focus on long-term outcomes.

##### BlephEx

For patients with anterior blepharitis and/or keratinization of the lid margin, therapies that clean the eyelid margin may be helpful. BlephEx (RySurg, Fort Worth, FL, USA) utilizes a rapidly spinning micro brush combined with a foam cleanser along the eyelid margin to remove encrusted secretions and other debris from the lid margin and eyelashes [63]. In a study of 81 patients with Demodex anterior blepharitis, individuals were randomized to receive one BlephEx or sham treatment in office, followed by 8 weeks of twice-daily 2% tea tree oil eyelid scrubs. Both groups showed improvement in OSDI, MGD grade, TBUT, and Demodex count 8 weeks after treatment. However, OSDI scores and Demodex count improved to a greater degree in the BlephEx group compared to sham (OSDI: 22.6 ± 8.2 vs. 27.1 ± 9.1, *p* = 0.03, respectively; Demodex Count: 2.6 ± 1.1 vs. 3.0 ± 1.3, *p* = 0.03, respectively) [64]. Overall, studies are limited by small sample sizes, short follow-up durations, and the variable use of comparison therapies, making it difficult to isolate the specific effects of in-office treatments, including BlephEx, on DED signs and symptoms. Additionally, the lack of direct comparisons to other eyelid treatments and inconsistencies in outcome measures highlight the need for larger, well-controlled trials to better define clinical efficacy in various disease populations.

#### 2.2.5. Periocular Skin-Based Treatments

##### Intense Pulsed Light (IPL) Therapy

MGD may be associated with dermatologic conditions such as rosacea and seborrheic dermatitis, which can contribute to eyelid inflammation, ocular surface disruption, and gland dysfunction [65]. Given its established use in treating rosacea, intense pulsed light (IPL) has been explored as a potential therapy for treating other periocular skin conditions and MGD [66]. IPL utilizes brief pulses of light to heat the periocular skin, perhaps exerting its effect by closing blood vessels that allow inflammatory cells and oxygen into the Meibomain glands [67,68,69]. In a study of 88 individuals with MGD-associated DED (MGS ≤ 12, TBUT ≤ 7 s, >5 non-atrophied glands in the lower eyelid, OSDI ≥ 23), patients were randomized to four IPL plus manual expression sessions versus manual expression alone. TBUT improved to a greater degree in the study versus the control arm (IPL: 4.0 ± 0.2 to 6.0 ± 0.3 vs. control: 3.8 ± 0.2 to 4.5 ± 0.3 s, *p* < 0.01). Symptoms also improved to a greater degree with IPL compared to manual expression alone (change in eye dryness on 0–100 VAS scale from baseline to four weeks after fourth treatment session IPL: −33.0 ± 2.6 vs. control: −22.1 ± 3.0, *p* = 0.0001) [70]. These findings suggest that IPL can be considered as an adjunctive therapy for MGD-associated DED in the appropriate patient population (e.g., those with skin rosacea).

#### 2.2.6. Oral Therapies

##### Omega-3 Fatty Acid Supplementation

Dietary supplementation, particularly with omega-3 fatty acids, has been investigated as a potential therapy for MGD with proposed anti-inflammatory properties and potential to change meibum composition [71]. Despite mixed results, some studies suggest that high-dose oral omega-3 supplementation may help tear film stability [72,73]. One study of 50 individuals with moderate MGD-associated DED (gland expression score 8–12, scale 0–24) randomized patients to a daily supplement (600 mg eicosapentaenoic acid and 1640 mg docosahexaenoic acid) or placebo (3000 mg olive oil) group. Improvement in TBUT was greater in the supplement compared to placebo group eight weeks after enrollment (4.1 ± 0.9 to 6.0 ± 1.6 s vs. 3.8 ± 0.8 to 5.1 ± 1.3 s, *p* = 0.03, respectively). Symptom improvement was observed in both groups, with no significant difference between groups [74]. The benefit of omega-3 was not replicated in a larger Dry Eye Assessment and Management (DREAM) study, which randomized 499 individuals with DED symptoms (OSDI 21–80, ATs ≥ 2 per day) to a daily dose of active supplement (3000 mg fish-derived *n*–3 eicosapentaenoic and docosahexaenoic acids) or placebo (olive oil) for 12 months. No significant differences in tear film stability (TBUT: 0.2 s, 95% CI −0.1–0.5) or DED symptoms (OSDI: −13.9 ± 15.6 vs. −12.5 ± 18.2) were found between groups. Additionally, improvements in conjunctival and corneal staining and tear production were similar across groups [75]. Although the findings have been mixed, omega-3 supplementation remains a therapeutic option in MGD-associated DED. While larger studies like DREAM did not demonstrate a significant benefit, positive results in smaller trials suggest that certain patient populations may still derive meaningful improvements, warranting further investigation into optimal dosing, formulation, and patient selection.

##### Oral Antibiotics

Certain antibiotics, such as doxycycline and azithromycin, have been studied in MGD due to their proposed anti-inflammatory and antimicrobial properties [76]. In a randomized control trial, 137 patients with moderate-to-severe MGD (based on the international workshop of MGD [77]) were assigned to receive doxycycline (200 mg daily for 6 weeks, *n* = 69) or azithromycin (1 g weekly for 3 weeks, *n* = 68). Both groups showed a similar reduction in mean MGD scores (6 weeks change from baseline (mean, standard error) azithromycin: −5.3, 0.5, *p* < 0.001; doxycycline: −5.0, 0.5, *p* < 0.001), which was similar between the two treatment groups. OSDI scores also improved to a similar degree in both groups (6 weeks change from baseline (mean, standard error) azithromycin: −4.8, 1.5, *p* = 0.001; doxycycline: −3.6, 1.5, *p* = 0.02) [78]. Other studies investigated the impact of different antibiotic dosing regimens on MGD. In a randomized control trial, 150 individuals with MGD (MGD grade ≥ 2, persistent symptoms ≥ 2 months with at-home treatments) were assigned to 200 mg doxycycline twice daily (high dose) versus 20 mg doxycycline twice daily (low dose) versus placebo. Both the high- and low-dose groups had significant improvements in TBUT, Schirmer test, and the number of symptoms 1 month after treatment compared to placebo, with no significant differences between dose groups [79]. These findings support the role of oral antibiotics in some individuals with MGD, though further research is needed to refine dosing strategies, evaluate long-term efficacy, and consider antibiotic side effect profiles.

### 2.3. Therapies Targeting Inflammation

Much of the existing data on DED posits inflammation as a core component of disease pathogenesis [80]. Ocular surface inflammation in DED involves an interplay between innate and adaptive immune responses, with both local and systemic inflammatory mediators contributing to disease progression [81,82]. As a result, targeting inflammation has become a central strategy in DED management. Measuring inflammation is a key component of the diagnostic workup in DED. Point-of-care tests such as InflammaDry detect the presence of MMP-9, an inflammatory marker, in the tears. Other imaging tests including IVCM can detect inflammation via the presence of activated dendritic cells, a biomarker of active inflammation [83]. When considering anti-inflammatory therapies, patient history and comorbidities should also be evaluated. Patients with systemic autoimmune diseases (i.e., Sjögren’s disease, graft-versus-host disease, Rheumatoid Arthritis) may benefit from long-term topical anti-inflammatory therapies such as cyclosporine and lifitegrast. Contrarily, patients with local inflammation such as rosacea may benefit from a short-term course of topical corticosteroids or oral anti-inflammatory therapies such as doxycycline and azithromycin, which offer potential additional benefits for MGD, as mentioned above [84]. Additionally, topical agents such as autologous serum tears (ASTs) and plasma rich in growth factors (PRGF) provide biologically derived anti-inflammatory and regenerative components that may support ocular surface healing and modulate immune activity in DED [85]. Below are several treatment approaches designed to reduce inflammation and improve ocular surface health in patients with DED.

#### 2.3.1. Topical Anti-Inflammatories

**Figure 3:**
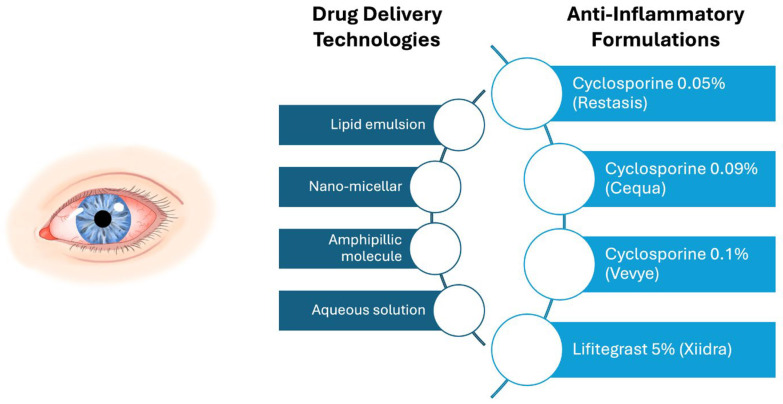
A comparison of FDA-approved topical anti-inflammatory therapies for dry eye disease, highlighting drug concentration and drug delivery vehicle. This schematic compares four anti-inflammatory eye drops approved for the treatment of dry eye disease: Restasis (cyclosporine 0.05%, oil-based emulsion), Cequa (cyclosporine 0.09%, nano-micellar solution), Vevye (cyclosporine 0.1%, semifluorinated alkane solution), and Xiidra (lifitegrast 5%, aqueous solution). This figure illustrates each drop’s active ingredient, drug concentration, and delivery technology. The vehicle type is annotated to emphasize differences in formulation strategy.

Topical anti-inflammatory therapies, including cyclosporine in multiple formulations and lifitegrast, target the underlying inflammatory pathways of DED by modulating immune responses on the ocular surface to reduce chronic inflammation and improve symptoms (Figure 3) [86]. Cyclosporine (CsA) is available in the United States in 0.05%, 0.09%, and 0.1% formulations (marketed as Restasis, Allergan, Dublin, Ireland; Cequa, Sun Pharmaceuticals, Cranbury, NJ, USA; and Vevye, Harrow Eye, Nashville, TN, USA, respectively). Widely used as an immunomodulatory therapy in other disease states, CsA reduces ocular surface inflammation in DED primarily by inhibiting calcineurin, thereby suppressing T-cell activation and pro-inflammatory cytokine release, which helps prevent ocular surface epithelial cell damage and apoptosis [87]. Multiple clinical studies have examined the efficacy of CsA. In a phase 3 study of individuals with DED (CFS ≥ 2, Schirmer ≤ 5), 293 received CsA 0.05% and were compared to 292 individuals who received a vehicle. When looking at symptoms, improvements from baseline to 6 months in dryness, sandy/gritty sensations, and itching were found in both groups, with no significant difference between groups. With respect to signs, CFS improved to a greater degree in the treatment versus vehicle group at 6 months (approximate mean change −0.88 vs. −0.67, *p* = 0.008) [88]. The most frequently reported adverse event in the treatment group in this study was mild ocular burning (14.7%, *n* = 47) and stinging (3.4%, *n* = 10). Over time, differing concentrations and topical delivery methods of CsA have been investigated. CsA 0.09% outcomes were similar to those of CsA 0.05%, with improvements in signs (i.e., Schirmer test and CFS) but not symptoms compared to the vehicle [89]. CsA 0.1%, the newest CsA formulation, was designed to enhance bioavailability and tolerability [90]. In a phase 3 study, 843 individuals with DED (CFS score ≥ 10, total conjunctival staining score ≥ 2, Schirmer 1–10 mm, dryness score ≥ 50 on VAS 0–100, and current AT use) were randomized to CsA 0.1% (*n* = 423) or vehicle (*n* = 411). Again, and similar to prior studies, the CFS score improved to a greater degree in the CsA 0.1% group compared to the vehicle at day 29 (LS mean group difference −0.4, 95% CI −0.8–0.0, *p* = 0.03). However, dryness scores showed similar improvements in both groups. Importantly, drop tolerability was excellent, with mean comfort scores ≤ 2.5 (scale 0–10) in both groups [90]. Given consistent improvements in signs more so than symptom relief, CsA-based topical anti-inflammatory agents may be the most effective when used in combination with adjunctive therapies targeting symptoms.

Lifitegrast 5% (Xiidra, Novartis Pharmaceuticals) is another topical anti-inflammatory therapy for DED that targets inflammation through lymphocyte function-associated antigen-1 (LFA-1) inhibition, offering an alternative to cyclosporine-based treatments [91]. In the initial phase 2 and OPUS-1 phase 3 clinical trials of individuals with mild-to-moderate DED (CFS ≥ 2 in one region, presence of conjunctival redness, Schirmer 1–10 mm, AT use within the past 6 months), improvement in DED signs was noted [92,93]. In the later OPUS-2 and OPUS-3 trials of individuals with moderate-to-severe DED (CFS ≥ 2 in one region on a 0–4 scale, conjunctival redness score ≥ 1 on a 0–4 scale, Schirmer 1–10 mm, eye dryness score ≥ 40 on a 0–100 scale), improvements in symptoms were identified [94,95]. The shift in inclusion criteria and outcome measures across the OPUS trials—from signs in earlier studies to symptoms in later ones—highlights the importance of defining the target population when evaluating outcomes. In a meta-analysis of 10 studies on lifitegrast, improvements were found in the total CFS score, TBUT, and ocular discomfort and eye dryness scores across various populations, with instillation site irritation and dysgeusia identified as the most common adverse effects [96]. Given these findings, lifitegrast may serve as an alternative for patients who may not respond adequately to CsA.

While CsA and lifitegrast both target inflammation, missing from the literature is a direct comparison of their effects. One study, however, examined 64 individuals who used both agents over the course of their DED journey and found that while the majority (51.6%) favored cyclosporine, a minority (21.9%) favored lifitegrast. This study could not identify demographics, comorbidities, or ocular surface findings that predicted medication preference [97], underscoring the challenge of identifying which individuals need a chronic anti-inflammatory therapy and which specific therapy is the best for each patient.

#### 2.3.2. Topical Corticosteroids

Topical corticosteroids, such as prednisolone, dexamethasone, fluorometholone (FML), and loteprednol, have been investigated as a short-term, fast-acting treatment option for inflammation in DED. These agents are typically considered in patients with significant ocular surface inflammation on clinical examination (e.g., marked conjunctival hyperemia or corneal staining), during acute symptom flares, or as bridging therapy when initiating slower-acting topical anti-inflammatories such as CsA or lifitegrast [98,99]. A meta-analysis of eight studies including 425 individuals with DED treated with topical corticosteroids found improvements in Schirmer scores and corneal staining, with a mean follow-up of 10.0 ± 15.3 weeks. Compared to control groups, topical corticosteroids also demonstrated improvement in TBUT [100]. Building upon these aggregate findings, individual randomized trials have further characterized the specific benefits of short-term steroid use in DED. A randomized control trial of 41 patients with DED (total CFS ≥ 1, TBUT ≤ 7 s, Schirmer < 10 mm, OSDI ≥ 12) assigned individuals to receive either 0.1% FML or topical polyvinyl alcohol (control) four times daily for 22 days. The FML group showed a significant reduction in CFS at day 21 compared to control (mean change −1.47, 95% CI −1.89–−1.05 vs. 0.00, 95% CI −0.14–0.14, *p* = 0.0008) [101]. These findings support the short-term use of topical corticosteroids as effective agents for improving ocular surface signs in DED, particularly in inflammation-driven cases. However, clinicians should be mindful of potential adverse effects associated with corticosteroid use, such as increased intra-ocular pressure and the risk of cataract formation, especially with prolonged use [102].

### 2.4. Therapies Targeting Nerves

**Figure 4:**
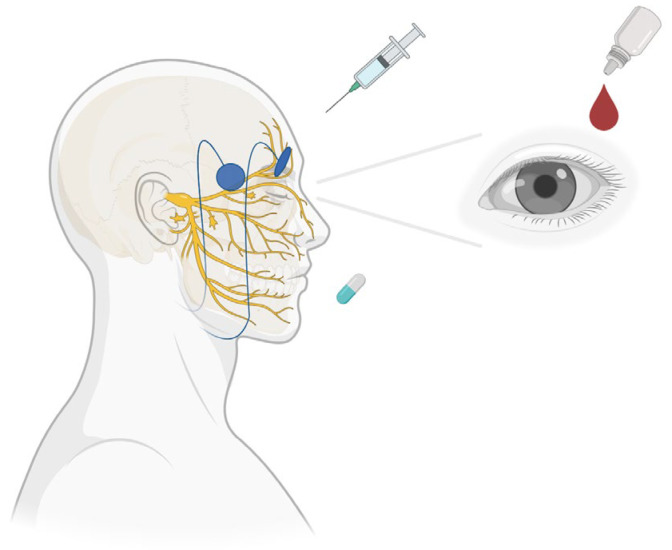
Nerve-targeted therapeutic strategies in dry eye disease. Therapies targeting nerve dysfunction in dry eye disease, organized by their primary site of action: ocular surface, peripheral nerves, and central nervous system. Interventions include topical therapies (e.g., autologous serum tears) acting on the ocular surface, adjuvant therapies (peripheral trigeminal transcutaneous electrical nerve stimulation (TENS) and botulinum toxin) targeting peripheral pathways, and oral neuromodulators targeting central pain processing.

Nerve dysfunction can contribute to DED pathophysiology by influencing tear production and function, ocular surface integrity, and ocular pain perception. As mentioned above, a thorough clinical exam including corneal sensation and proparacaine challenge test and an imaging test such as IVCM, may indicate nerve contributors to DED. Corneal nerve damage characterized by the loss of sensation and associated corneal pathology, termed neurotrophic keratitis (NK), can lead to tear film disruption and ocular surface instability. While nociceptive pathways mediate physiological pain response to insults such as tear hyperosmolarity and ocular surface damage, neuropathic and nociplastic mechanisms may also be underlying drivers of symptoms that are often disproportionate to clinical signs. Importantly, the diagnostic framework for nerve-mediated (neuropathic, nociplastic) DED continues to evolve, incorporating both symptom-based assessments and objective testing. Clinical evaluation often includes symptom descriptors such as burning, shooting, or sensitivity to wind and light, along with diagnostic adjuncts like the proparacaine challenge and IVCM. Although diagnostic tools are improving, nerve dysfunction in DED often presents alongside other causes of ocular surface discomfort such as aqueous deficiency or Meibomian gland dysfunction. This overlap complicates both accurate diagnosis and appropriate treatment selection. Therapies targeting nerves aim to either restore nerve function and/or modulate nerve signaling. Below are several therapeutic approaches, including oral and topical pharmacologic interventions and neurostimulation devices (Figure 4).

#### 2.4.1. Blood Products

Autologous serum tears (ASTs) and plasma rich in growth factors (PRGF) have been studied as treatments in various DED subtypes, including those with nerve dysfunction. These blood-derived biologic therapies contain growth factors, anti-inflammatory cytokines, and bioactive components that mimic the natural composition of tears, supporting epithelial regeneration, targeting nerve dysfunction, and modulating immune responses [103]. When considering these biologic agents, it is important to note that ASTs, as a blood product, are not FDA-approved and are instead prepared under sterile compounding protocols at individual pharmacies or institutional blood banks, resulting in variability in formulation, dilution, and storage procedures. In addition, cost, a lack of insurance coverage, and limited availability present significant barriers. ASTs require phlebotomy, specialized laboratory processing, and strict refrigeration, which restrict access primarily to patients living near academic centers or tertiary care facilities equipped to provide such services. These limitations must be considered when weighing the potential benefits of ASTs in patients with refractory or neurotrophic DED.

Autologous serum tears (ASTs) are usually prepared in serum concentrations ranging from 20 to 50% [104]. Several reviews have highlighted inconsistencies in current data on the benefits of AST use in DED [105,106]. However, benefits have been suggested across multiple studies. In a meta-analysis of 12 randomized control trials of AST versus artificial tear use in DED, improvements in Schirmer test scores (weighted mean difference (WMD)  =  2.35, 95% CI 1.45–3.24, *p* < 0.001), TBUT (WMD  =  2.83, 95% CI 2.27–3.39, *p* < 0.001), CFS (standard mean difference (SMD)  =  −2.11, 95% CI −3.07–−1.15, *p* < 0.001), and OSDI (WMD  =  −10.54, 95% CI −13.31–−7.77, *p*  <  0.001) were noted [107]. Building on these findings, one randomized controlled trial offered further insight into AST efficacy. In a randomized control trial of 230 individuals with DED (TBUT < 5 s, Schirmer < 5 mm, CFS ≥ 2, OSDI ≥ 23), participants were assigned to 20% AST (*n* = 116) or artificial tears (*n* = 116) four times daily for 12 weeks. While TBUT, Schirmer, CFS, conjunctival impression cytology (CIC), and OSDI scores improved in both groups at 12 weeks, the AST group showed greater improvements in all outcomes (TBUT: 3.1 s, 95% CI 2.3–3.9, *p* < 0.05; Schirmer: 1.8 mm, 95% CI 1.2–2.4, *p* < 0.05; CFS: −1.5, 95% CI −1.9–−1.1, *p* < 0.05; CIC: −0.7, 95% CI −0.9–−0.5, *p* < 0.05; OSDI: −10.3, 95% CI −13.6–−7.0, *p* < 0.001) [108]. Other formulations of blood-derived eye drops, such as plasma rich in growth factors (PRGF) and platelet-rich plasma (PRP), have also shown efficacy in open-label studies [109]. These data highlight that blood products may have a role in managing DED signs and symptoms, although the optimal population and formulation in which they should be used need better definition.

Given the role of nerve dysfunction in the pathogenesis of NK, ASTs have also been studied as a treatment for NK [110]. In an open-label study of 11 eyes of six individuals with grade 1 or 2 NK, patients were treated with topical AST 6–8 times daily for 1 month, tapered to 4 times daily. This study reported improvement in corneal staining, corneal sensitivity, and symptom scores after treatment (CFS: 6.1 ± 4.6 to 1.6 ± 4.3, *p* = 0.0003; corneal sensitivity (Cochet-Bonet): 0.9 ± 1.2 to 4.2 ± 1.4, *p* < 0.0001; OSDI: 39.5 ± 10.2 to 16.8 ± 6.0, *p* = 0.003). This study also identified an increase in nerve number and length after treatment on in vivo confocal microscopy, highlighting the potential nerve regeneration benefits of ASTs in NK (number: 0.8 ± 0.8 to 4.8 ± 2.0, *p* = 0.0004; length: 136.7 ± 130.0 μm to 1362.6 ± 559.0 μm, *p* = 0.0002) [111]. Although encouraging, more robust methodologies are needed to examine the impact of ASTs and other blood products on NK. Due to the potential nerve regeneration properties of ASTs, they have also been used in the treatment of peripheral neuropathic pain, but randomized control data is limited [26].

#### 2.4.2. Oral Neuromodulators

In patients with systemic pain comorbidities (i.e., fibromyalgia, migraine) or refractory pain symptoms after the optimization of the ocular surface and evidence of central abnormalities (persistent pain after anesthetic eye drops, pain characteristics of wind and light hypersensitivity), oral neuromodulators may be an appropriate next step in therapy [12,26]. Oral neuromodulators act on pain pathways to modulate neuronal excitability and reduce hyperalgesia. Several drug classes have been investigated for this purpose, including α2δ ligands (gabapentin, pregabalin), tricyclic antidepressants (TCAs) such as nortriptyline and amitriptyline, serotonin–norepinephrine reuptake inhibitors (SNRIs) like duloxetine, and anticonvulsants (carbamazepine, oxcarbazepine, and topiramate) (Table 2). Below, we review the available evidence on the efficacy and potential role of these agents in managing chronic ocular pain associated with DED.

Gabapentin and pregabalin, both α2δ ligands, have been investigated for their potential in treating neuropathic ocular pain (NOP) due to their action on calcium channels, which leads to a reduction in excitatory neurotransmitter release [113]. In an open-label study of 72 individuals with DED (TBUT < 5 s, Schirmer < 5 mm, ocular symptoms > 6 months), all patients received artificial tears and cyclosporine drops for 6 weeks. A total of 36 patients who had DED with neuropathic pain features (painDETECT questionnaire (PD-Q) > 18) were additionally given gabapentin 600–2400 mg/day for 6 weeks. After 6 weeks of treatment, both groups had an improvement in TBUT, Schirmer scores, and OSDI, with significant differences between the non-gabapentin- and gabapentin-treated groups (TBUT: 9.9  ±  1.7 s vs. 12.8  ±  2.0 s, *p* < 0.001; Schirmer 10.1  ±  2.6 mm vs. 14.2  ±  3.0 mm, *p* < 0.001, OSDI 49.4  ±  16.7 vs. 31.1  ±  11.5, *p* < 0.001) [118]. These results suggest that adjunctive gabapentin may enhance both subjective and objective treatment outcomes in patients with DED exhibiting neuropathic pain features. It is also important to note that these medications can take up to 3 months to show a therapeutic effect and can have undesirable side effects including drowsiness, dizziness, and ataxia that often get better with time [116]. Therefore, careful titration and a sufficient treatment period, typically 3 to 6 months, are essential for achieving therapeutic benefit. In conclusion, the available data on the efficacy of gabapentin and pregabalin in managing dry eye disease and ocular pain is mixed and sparse.

Originally developed as antidepressants, tricyclic antidepressants (TCAs) such as nortriptyline and amitriptyline have been widely used for non-ocular neuropathic pain and migraine. Their analgesic effects, attributed to serotonin and norepinephrine reuptake inhibition and sodium channel blockade, have led to their investigation as potential treatments for ocular pain [114,119]. With regard to ocular pain, a retrospective study of 30 individuals with persistent symptoms (chart diagnosis of ocular pain and continued pain after topical anesthesia) evaluated the effects of nortriptyline use (10–100 mg daily) for at least four weeks. The study found a significant reduction in the average 24 h ocular pain scores from baseline to the final visit (Ocular Pain Assessment Survey (OPAS): 5.7 ± 2.1 to 3.6 ± 2.1, *p* < 0.0001). However, despite symptom improvement, 26.6% (*n* = 8) of patients discontinued treatment due to side effects [114]. While oral neuromodulators offer a potential avenue for managing ocular pain, their use requires careful patient selection and monitoring due to variable efficacy and tolerability.

#### 2.4.3. Adjuvant Therapies

##### Botulinum Toxin

In patients with features of light sensitivity, therapies that have been used to target other diseases with light sensitivity (e.g., migraine) can be considered. Botulinum toxin has been used in this regard based on its effect on light sensitivity in migraine [116,120]. In a retrospective study, 27 individuals with features of NOP received botulinum toxin injections based on a modified migraine protocol. A total of 74.1% (*n* = 20) reported at least some pain improvement 1 month after injection [121]. These preliminary findings suggest that botulinum toxin may offer benefit for patients with certain ocular pain features, but more robust randomized controlled trials are needed to define its therapeutic role and identify ideal candidates for treatment.

##### Peripheral Trigeminal Transcutaneous Electrical Nerve Stimulation (TENS)

TENS has been explored as a treatment modality for various chronic pain conditions, including fibromyalgia and migraine, with studies indicating potential benefits in pain reduction [122,123]. Given these positive outcomes, TENS has been investigated for its efficacy in managing ocular pain, especially in individuals with light sensitivity and/or migraine. In a randomized controlled trial of 52 individuals with DED (Schirmer < 10 mm, TBUT < 10 s, OSDI ≥ 13), participants were assigned to either TENS (Huatuo brand SDZ-II Electrical Stimulator, China) five sessions per week (with electrodes placed on the skin targeting the ophthalmic (V1) and maxillary (V2) branches of the trigeminal nerve) along with artificial tears (ATs) four times daily (*n* = 26) or artificial tears alone (*n* = 26) for 4 weeks. OSDI scores decreased in both groups, with a significant difference between the TENS + AT and AT groups at the end of 4 weeks (42.3 ± 7.6 to 24.5 ± 4.8 vs. 43.2 ± 6.2 31.3 ± 8.6, *p* = 0.004, respectively). Additionally, TENS + AT treatment resulted in greater improvements in TBUT, Schirmer scores, and CFS compared to artificial tears alone [124]. These findings suggest that TENS may provide therapeutic benefits for ocular pain and may also impact tear parameters. Further research, including randomized controlled trials, are needed to better define efficacy, optimal patient selection, device parameters, and long-term outcomes.

## 3. Conclusions

DED is a multifactorial condition with complex pathophysiology, necessitating a diverse array of treatment strategies targeting tear film instability, ocular surface inflammation, and nerve dysfunction, in the appropriate patient. Given the heterogeneity of DED presentations, a multimodal treatment approach is essential for optimizing patient outcomes. The landscape of DED therapeutics has expanded significantly and now includes artificial tears, therapies that target eyelid health and Meibomian glands, anti-inflammatory agents, regenerative treatments, and neuromodulatory agents, to name a few. However, selecting the right agent requires matching it to the right patient, guided by the clinical presentation and the underlying mechanisms driving signs and symptoms. While emerging studies have begun to explore which therapies may be the most effective for specific subgroups of patients with DED and ocular pain, many current investigations remain limited by open-label designs, a lack of direct comparisons between active agents, and an insufficient consideration of underlying differences in nerve function. Refining therapeutic approaches will require well-controlled, mechanism-driven studies that account for the heterogeneity of pain phenotypes and guide personalized treatment strategies.

Despite advances in DED management, gaps remain in understanding the most effective treatment combinations, long-term outcomes, and the interplay between nociceptive and neuropathic mechanisms in individual patients. Future research should focus on personalized medicine approaches, integrating biomarkers, imaging techniques, and predictive models to guide targeted, mechanism-based treatment strategies for patients with DED and ocular pain.

## Figures and Tables

**Table 1:** Overview of therapies reviewed for dry eye disease. Reported outcomes are based on group-level findings from clinical studies. Treatment responses are heterogeneous and may differ among individuals, even within the same DED subtype.

Therapy	Type of DED Targeted	Proposed Mechanism of Action	Key Clinical Outcomes
Artificial tears	ATD/MGD	Supplement aqueous layer of tear film and/or improve tear film stability using lipid-based components	Improved signs and symptoms across multiple formulations and trials in ATD and MGD-associated DED
Punctal plugs	ATD	Augment ocular surface tear retention	Improved tear retention in some studies and improved symptoms; evidence variable
Tyrvaya (varenicline Solution)	ATD	Stimulate tear production via trigeminal pathway	Increased tear production, variable improvement in symptoms versus placebo
External nasal stimulation	ATD	Stimulate external nasal nerves to promote tear production	Increased tear production after regular use
Perfluorohexyloctane ophthalmic solution (MEIBO)	MGD	Improve tear film function	Improved corneal staining and symptoms in patients with MGD; no significant effect on MGD score or TBUT versus placebo
Eyelid hygiene	MGD	Reduce debris and bacteria at lid margin	Limited change in gland function or signs; improved symptoms
LipiFlow	MGD	Heat and pulsation to evacuate Meibomian glands	Improved gland function, tear film stability, and symptoms versus warm compress; treatment durability varied across studies
iLux	MGD	Targeted heat and compression to clear gland obstructions	Improved gland function, tear film stability, and symptoms versus manual gland expression; comparable efficacy to LipiFlow; variable overall efficacy across studies
BlephEx	MGD	Mechanical cleaning of lid margin	Improved Demodex count and symptoms versus sham treatment; limited comparative data
IPL	MGD	Reduce inflammation and improve gland function	Improved tear stability and symptoms in MGD with skin inflammation versus manual gland expression alone
Omega-3 supplementation	MGD	Anti-inflammatory effect; may alter meibum composition	Mixed findings; some studies showed symptom and tear stability benefit, while others did not
Oral antibiotics	MGD/Inflammation	Reduce inflammation and bacterial load	Improved gland function and symptoms in MGD across several regimens
Topical anti-inflammatories	Inflammation	Suppress ocular surface inflammation (e.g., CsA, lifitegrast)	Improved corneal staining across all studies; variable improvement in other signs and symptoms versus vehicle
Topical corticosteroids	Inflammation	Suppress ocular surface inflammation	Short-term improvement in signs and symptoms in inflammation-driven DED versus control
Blood products	Nerves	Provide growth factors and anti-inflammatory cytokines	Improved signs and symptoms compared to artificial tears, strongest signal in moderate or greater DED and neurotrophic keratitis-related DED
Oral neuromodulators	Nerves	Modulate central pain signaling pathways	Reduced ocular pain symptoms in some patients with neuropathic features
Botulinum toxin	Nerves	Modulate nerve activity	Improved ocular pain in some patients with neuropathic features; limited data overall
Peripheral TENS	Nerves	Stimulate trigeminal nerve branches to modulate pain	Improved symptoms versus artificial tears alone; variability in tear parameter improvement between devices and studies

**Table 2:** Overview and dosing of oral neuromodulators. All therapies need to be started at low doses and titrated slowly to therapeutic dose.

Drug	Mechanism	Dose Range Used in Individuals with Chronic Ocular Pain	Ocular Pain Studies
Gabapentin Pregabalin	α2δ voltage-gated calcium channel ligand	Gabapentin—300 mg–900 mg TID Pregabalin—75 mg–150 mg BID	Shen, X. et al. [112] Tei, Y. et al. [113]
Amitriptyline, Nortriptyline	Serotonin and norepinephrine reuptake inhibition, sodium channel modulation	Nortriptyline—10 mg–100 mg daily Amitriptyline—10 mg–100 mg daily	Ozmen, M.C. et al. [114]
Duloxetine	Selective norepinephrine reuptake inhibitor	20–60 mg daily	Small, L.R. et al. [115] Patel, S. et al. [116]
Topiramate	Voltage-gated sodium channel modulation, enhances GABA-A receptor activity	100 mg daily	Patel, S. et al. [116]
Low-Dose Naltrexone	Pure opioid antagonist with μ− and δ opioid receptor affinity	1.5–4.5 mg daily	Dieckmann, G. et al. [117]

## Abbreviations

The following abbreviations are used in this manuscript:

MDPI	Multidisciplinary Digital Publishing Institute
DED	Dry eye disease
ATD	Aqueous tear deficiency
MGD	Meibomian gland dysfunction
DEQ5	5-Item Dry Eye Questionnaire 5
OSDI	Ocular Surface Disease Index
TBUT	Tear break-up time
NK	Neurotrophic keratitis
ATs	Artificial tears
tCFS	Total corneal fluorescein staining
MGS	Meibomian gland secretion
IVCM	In vivo confocal microscopy
CsA	Cyclosporine
ASTs	Autologous serum tears
